# Publisher Correction: A square-root topological insulator with non-quantized indices realized with photonic Aharonov–Bohm cages

**DOI:** 10.1038/s41467-020-15252-6

**Published:** 2020-04-08

**Authors:** Mark Kremer, Ioannis Petrides, Eric Meyer, Matthias Heinrich, Oded Zilberberg, Alexander Szameit

**Affiliations:** 1 Institut für Physik, Universität Rostock, Albert-Einstein-Straße 23, 18059 Rostock, Germany; 2 Institut für Theoretische Physik, ETH Zürich, Wolfgang-Pauli-Straße 27, 8093 Zürich, Switzerland

**Keywords:** Micro-optics, Topological insulators, Quantum optics

Correction to: *Nature Communications* 10.1038/s41467-020-14692-4, published online 14 February 2020.

The original version of this article had an error in Fig. 4d. On the upper-left side of the equation “$E = \pm 2t$”, The “$\mp$” sign was mistakenly typed as a “$\pm$” sign. A correct Fig. 4 is supplemented with this correction. This error has been corrected in both the PDF and HTML versions of the article.

**Figure 1:**
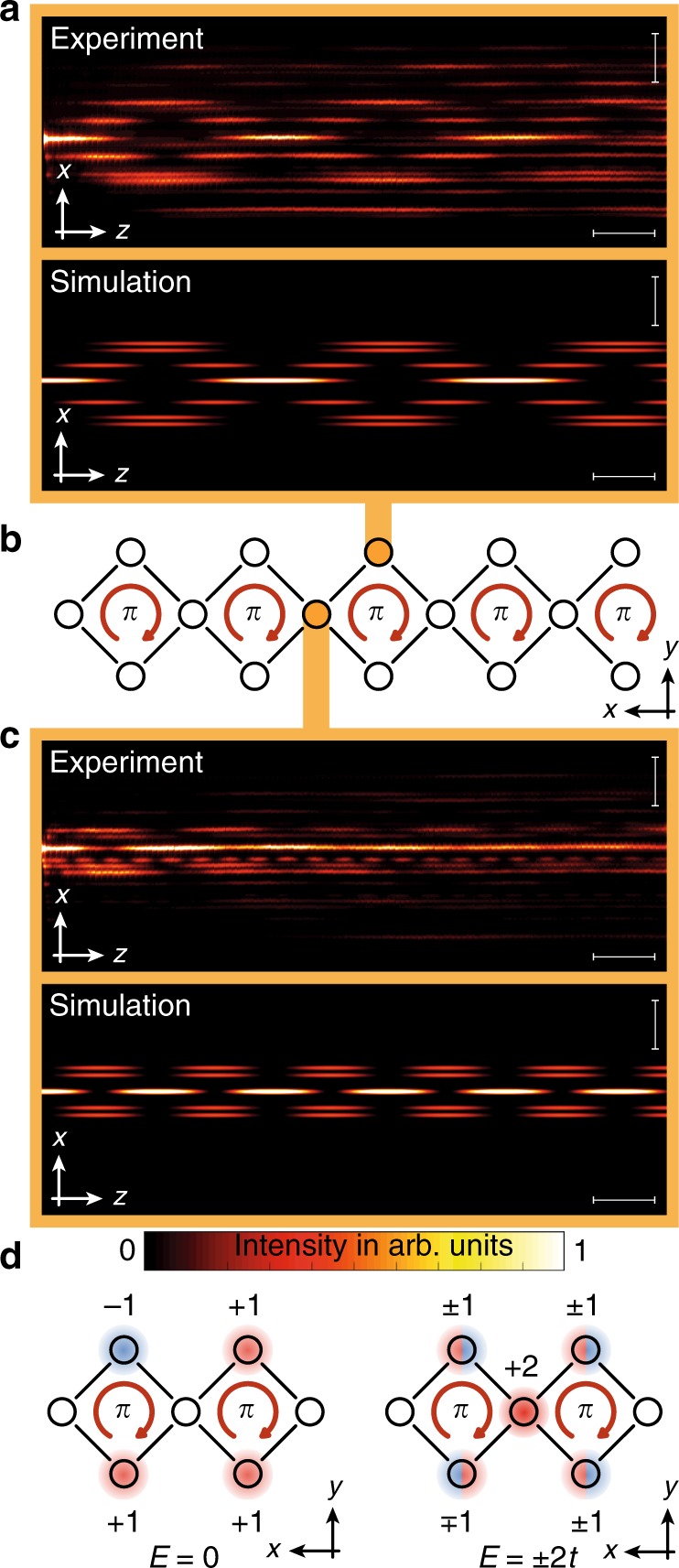
Fig. 4

