# Supplementary material for: A square-root topological insulator with non-quantized indices realized with photonic Aharonov-Bohm cages
Source: Nat Commun. 2020 Feb 14;11:907. doi: 10.1038/s41467-020-14692-4 (PMC7021779; doi:10.1038/s41467-020-14692-4)
Supplement: Supplementary file 1 — Supplementary Information [file 41467_2020_14692_MOESM1_ESM.pdf]

Supplementary Information for

A square-root topological insulator with non-quantised indices realised with photonic Aharonov-Bohm cages

Mark Kremer[1], Ioannis Petrides[2,], Eric Meyer[1], Matthias Heinrich[1],
Oded Zilberberg[2], and Alexander Szameit[1]

[1] *Institut für Physik, Universität Rostock, Albert-Einstein-Straße 23, 18059 Rostock, Germany.*
[2] *Institut für Theoretische Physik, ETH Zürich, Wolfgang-Pauli-Straße 27, 8093 Zürich, Switzerland.*

SUPPLEMENTARY FIGURES

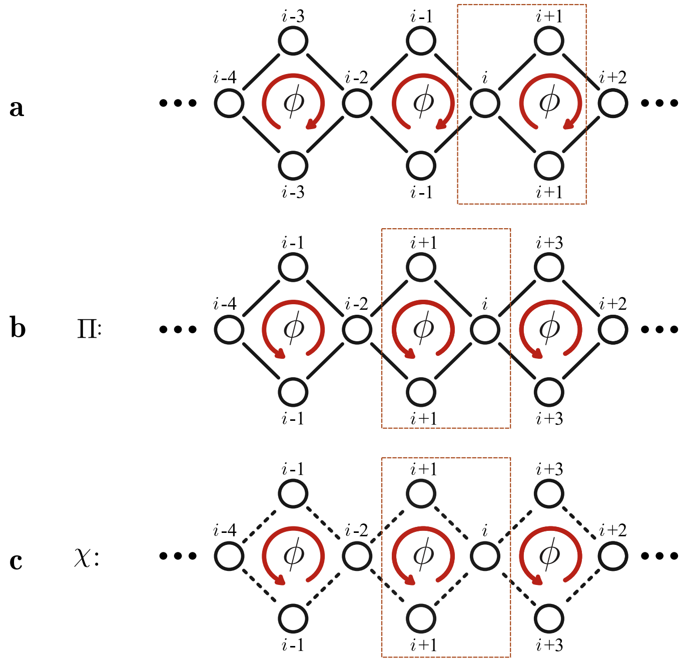

Supplementary Figure 1. The two non symmorphic symmetries, Π and χ, acting on an infinite chain of AB-cages with periodic boundary conditions. **a** The original lattice of AB-cages with flux ϕ threading each plaquette. **b** The lattice obtained after a Π transformation. **c** The lattice obtained after a χ transformation. The dashed box shows a chosen set of basis points. Solid (dashed) lines denote positive (negative) hopping strengths.

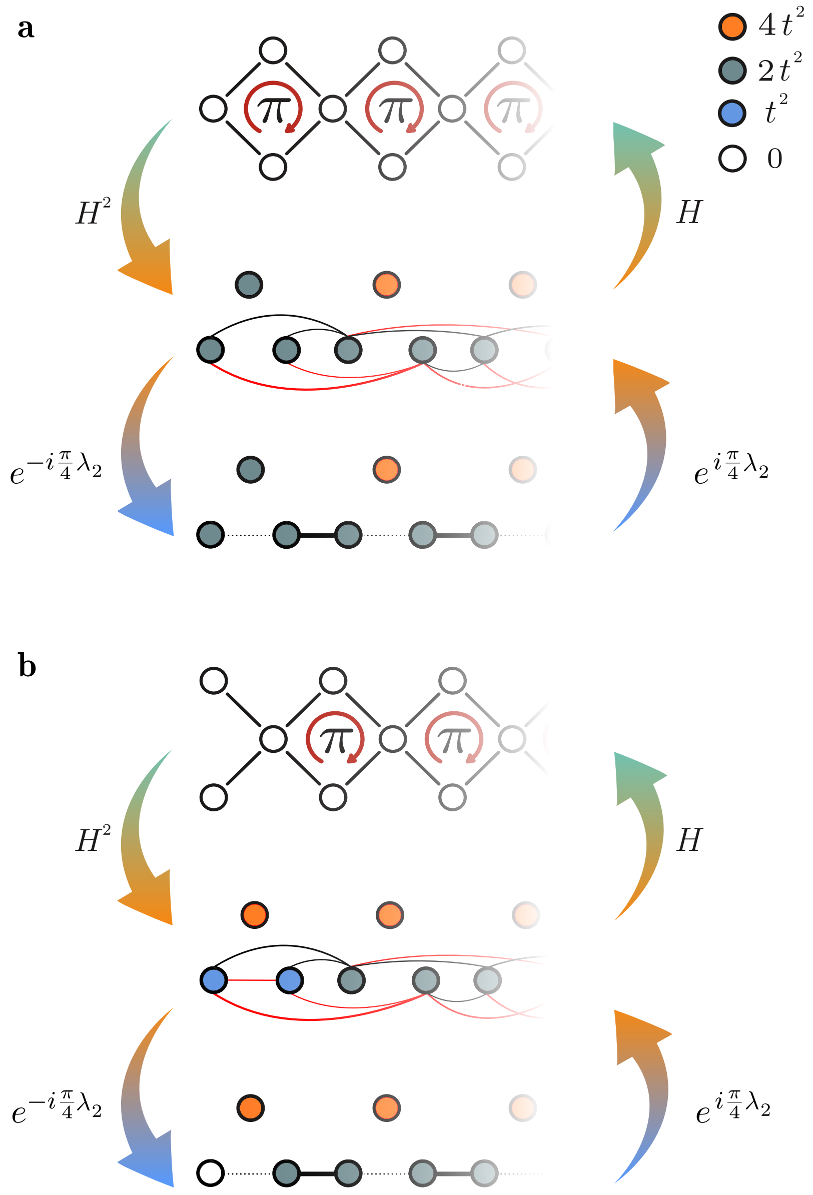

Supplementary Figure 2. Square mapping semi-infinite chains. **a** A semi-infinite chain of Aharonov-Bohm cages with a termination that supports two nontrivial topological in-gap boundary states [cf. Supplementary Eq. (10)]. The corresponding H^2 model exhibits at its boundary two in-gap degenerate states: one appears as a standard manifestation of the SSH model's bulk-boundary correspondence and the second belongs to the subspace of isolated states, now found at a lower energy [cf. Supplementary Eq. (18)]. **b** A semi-infinite chain of Aharonov-Bohm cages with a termination that does not support topological in-gap boundary modes [cf. Supplementary Eq. (12)]. The corresponding H^2 model similarly does not exhibit topological in-gap boundary states: the standard SSH model has a termination with corresponding boundary mode that is lowered to hybridize with the bulk band at zero energy [cf. Supplementary Eq. (21)].

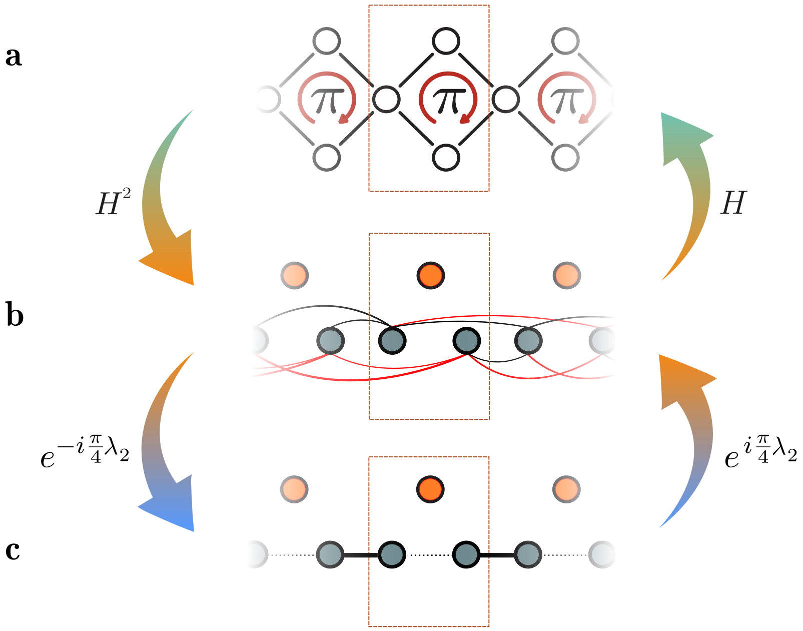

Supplementary Figure 3. Square mapping the infinite chain. **a** An infinite chain of Aharonov-Bohm cages with $\phi = \pi$ is mapped to **b** a chain with positive (black) and negative (red) next-to-nearest neighbour hopping and one decoupled state per unit cell. Rotating the basis with $\mathrm{e}^{-\mathrm{i}\frac{\pi}{4}\lambda_2}$, the latter is mapped to **c** the SSH model with an additional decoupled state per unit cell.

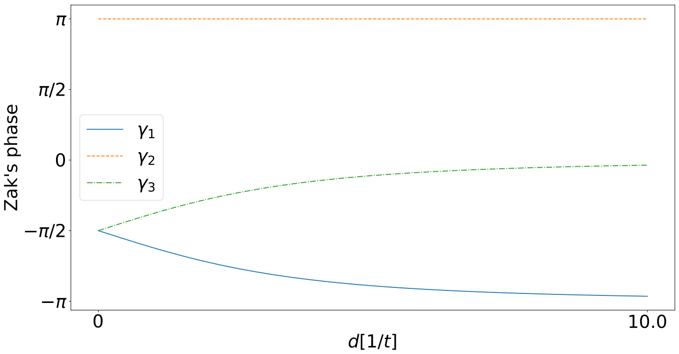

Supplementary Figure 4. Zak's winding phases of the bands of $H(k)$ [cf. Supplementary Eq. (32)] as a function of the on-site potential d. The winding of the zero-energy band γ_2 remains unaltered, whereas γ_1 and γ_3 are continuously mixed to the point where $\gamma_1 \to \pi$ and $\gamma_3 \to 0$.

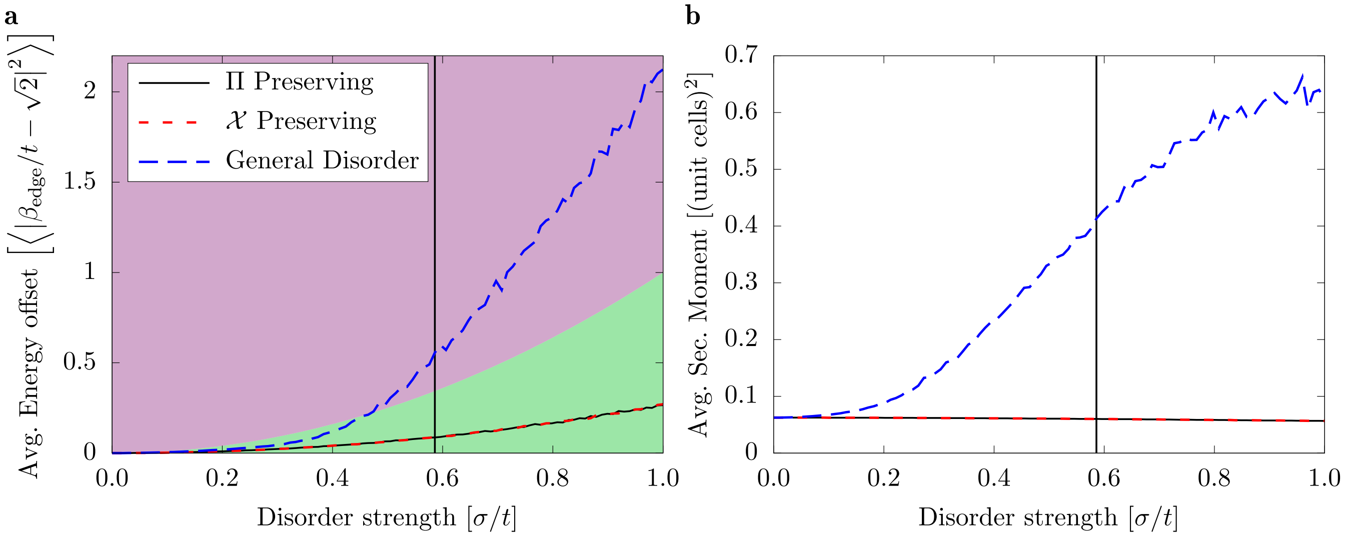

Supplementary Figure 5. **Disorder Analysis of Energy and Localisation.** **a** Disorder analysis of the energy offset of the topological edge state with on average preserved symmetries. The vertical axis shows the averaged mean squared difference $\langle|\beta_{\text{edge}}/t-\beta_0/t|^2\rangle$ of the edge state energy β_{edge} in the presence of disorder and the energy of the undisturbed system $\beta_0=\sqrt{2}t$. The vertical colored solid line indicates where the standard deviation of the disorder reaches the size of the gap from the boundary state to the nearest bulk band in each corresponding case. The red and green regions define an energy offset that is bigger (red) or smaller (green) than $(\sigma/t)^2$, which would correspond to a an energy offset proportional to the disorder strength.**b** The average second moment (localization length) of the eigenstate, corresponding to the eigenenergy of **a**. Each disorder type was realized to either preserve, or break certain symmetries, as labeled in the plots. The disorder-averaging simulations were run using a lattice with 99 sites and every disorder strength was realized 10000 times.

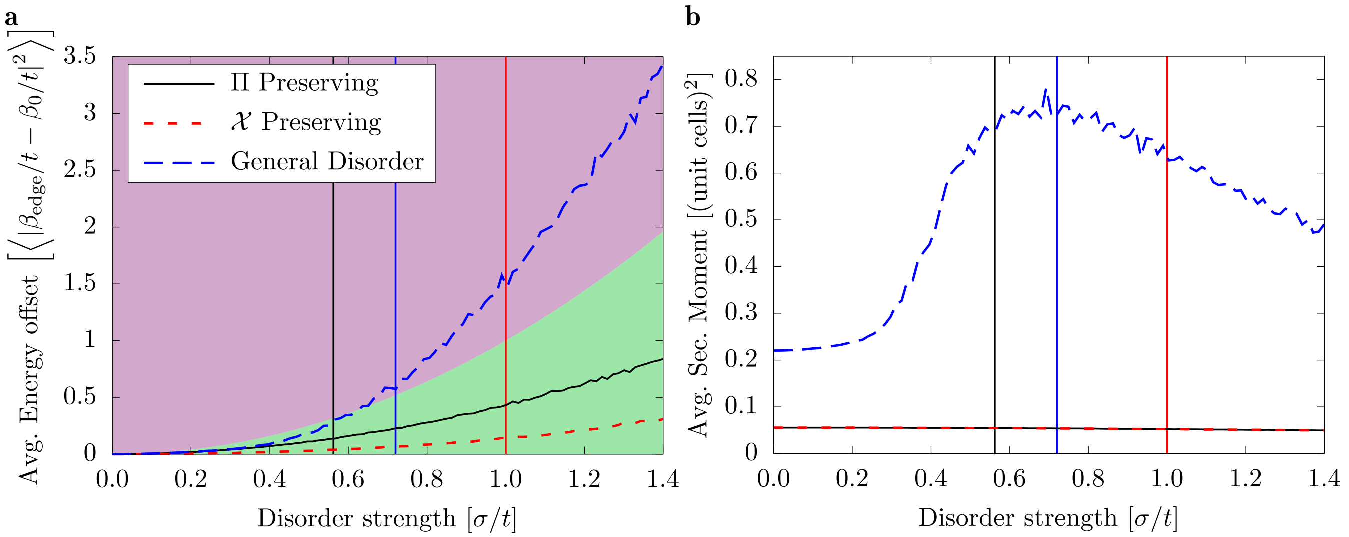

Supplementary Figure 6. **Disorder Analysis of Energy and Localisation.** **a** Disorder analysis of the energy offset of the topological edge state with on average broken symmetries. The vertical axis shows the averaged mean squared difference $\langle|\beta_{\text{edge}}/t-\beta_0/t|^2\rangle$ of the edge state energy β_{edge} in the presence of disorder and the energy of the undisturbed system β_0 (i.e., when $\sigma=0$). The vertical colored solid lines indicate where the standard deviation of the disorder reaches the size of the gap from the boundary state to the nearest bulk band in each corresponding case. The red and green regions define an energy offset that is bigger (red) or smaller (green) than $(\sigma/t)^2$, which would correspond to a an energy offset proportional to the disorder strength.**b** The average second moment (localization length) of the eigenstate, corresponding to the eigenenergy of **a**. Each disorder type was realized to either preserve, or break certain symmetries, as labeled in the plots. The disorder-averaging simulations were run using a lattice with 99 sites and every disorder strength was realized 10000 times.

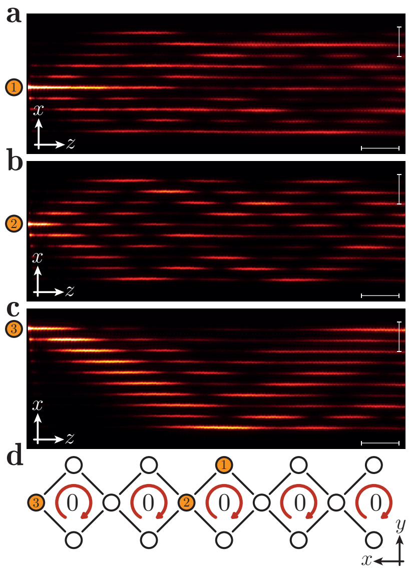

Supplementary Figure 7. **Propagation without Flux.** **a** Light dynamics when exciting the top site in a single bulk plaquette without flux (labeled as 1 in **d**). The horizontal scale bar corresponds to 1 cm, while the vertical scale bar corresponds to 50 μm. **b** Light dynamics when exciting the lattice site inbetween two bulk plaquettes without flux (labeled as 2 in **d**). The horizontal scale bar corresponds to 1 cm, while the vertical scale bar corresponds to 50 μm. **c** Light dynamics when exciting the edge lattice site of a lattice without flux (labeled as 3 in **d**). The horizontal scale bar corresponds to 1 cm, while the vertical scale bar corresponds to 50 μm. **d** Overview of which lattice sites are excited in **a**-**c**.

SUPPLEMENTARY NOTES

Supplementary Note 1

Here we discuss the Bulk properties of $H(k)$.The spectrum of $H(k)$ [cf. Eq. (1) in the main text] has three bands at energies $\pm t\sqrt{2}\sqrt{1+\cos k+\cos(k-\phi)}$ and 0, see Fig. 1a in the main text. When the flux threading the plaquettes is $\phi \neq 0 \bmod 2\pi$, the three bands are isolated from each other, therefore, an Abelian Zak's phase can be defined to each,

$$\gamma_i = \mathrm{i}\int_{-\pi}^{\pi} \mathrm{d}k \langle v_i(k)|\partial_k|v_i(k)\rangle \,, \tag{1}$$

where $i=1,2,3$ labels the bands in increasing energy. The bulk band solutions $|v_i(k)\rangle$ are found by diagonalizing the Hamiltonian $H(k)$ and can be written in a nonsingular, analytical form

$$|v_1\rangle = \frac{1}{2\sqrt{2}}\begin{pmatrix} -2\sqrt{2+\cos k+\cos(k-\phi)} \\ 1+\mathrm{e}^{\mathrm{i}k} \\ \mathrm{e}^{\mathrm{i}\phi}+\mathrm{e}^{\mathrm{i}k} \end{pmatrix}, \quad |v_2\rangle = \frac{1}{2}\begin{pmatrix} 0 \\ \mathrm{e}^{\mathrm{i}\phi}+\mathrm{e}^{\mathrm{i}k} \\ 1+\mathrm{e}^{\mathrm{i}k} \end{pmatrix}, \quad |v_3\rangle = \frac{1}{2\sqrt{2}}\begin{pmatrix} 2\sqrt{2+\cos k+\cos(k-\phi)} \\ 1+\mathrm{e}^{\mathrm{i}k} \\ \mathrm{e}^{\mathrm{i}\phi}+\mathrm{e}^{\mathrm{i}k} \end{pmatrix}. \tag{2}$$

Using Supplementary Eqs. (1) and (2), we find

$$\begin{aligned} \gamma_1 = \gamma_3 &= \frac{3\pi}{2} \bmod 2\pi \\ \gamma_2 &= \pi \bmod 2\pi \,. \end{aligned} \tag{3}$$

The winding phases of $H(k)$ [cf. Supplementary Eqs. (3)] are constrained by two nonsymmorphic symmetries (see Supplementary Figure 1), namely

$$\chi = \begin{pmatrix} 1 & 0 & 0 \\ 0 & -\mathrm{e}^{-\mathrm{i}k} & 0 \\ 0 & 0 & -\mathrm{e}^{-\mathrm{i}\phi}\mathrm{e}^{-\mathrm{i}k} \end{pmatrix} \text{ and } \Pi = \begin{pmatrix} 1 & 0 & 0 \\ 0 & \mathrm{e}^{-\mathrm{i}k} & 0 \\ 0 & 0 & \mathrm{e}^{-\mathrm{i}\phi}\mathrm{e}^{-\mathrm{i}k} \end{pmatrix}, \tag{4}$$

The above transformations act on the Hamiltonian in the following way

$$\Pi H(k)\Pi^{-1} = H^*(k)\,, \tag{5}$$

$$\chi H(k)\chi^{-1} = -H^*(k)\,, \tag{6}$$

where $H^*(k)$ is the conjugate Hamiltonian and the equalities are up to gauge transformations. These symmetries lead to the following relations between the winding phases of the bands:

$$\begin{aligned} \Pi: &\quad \gamma_1+\gamma_3 \in \{0,\pi\} \quad &\text{and}\quad &\gamma_2 \in \{0,\pi\}\,, \\ \chi: &\quad \gamma_1=\gamma_3 \in \{0,\pm\pi/2\} \quad &\text{and}\quad &\gamma_2 \in \{0,\pi\}\,. \end{aligned} \tag{7}$$

Breaking the χ symmetry with an onsite potential allows γ_1 and γ_3 to take any phase value, but, interestingly, their sum remains a $\mathbb{Z}_2$ invariant due to the Π symmetry(see Supplementary Note 6).

Supplementary Note 2

Here we discuss the boundary properties of H. To derive the explicit solutions of boundary states, we first consider a semi-infinite AB-cages chain, at $\phi=\pi$, with a termination shown in Supplementary Figure 2A. The real-space representation of the Hamiltonian is given by

$$H = t\begin{pmatrix} D & T & 0 & \cdots \\ T^\dagger & D & T & \ddots \\ 0 & T^\dagger & D & \ddots \\ \vdots & \ddots & \ddots & \ddots \end{pmatrix}, \tag{8}$$

where t is the hopping amplitude,

$$D = \begin{pmatrix} 0 & 1 & -1 \\ 1 & 0 & 0 \\ -1 & 0 & 0 \end{pmatrix}, \qquad \text{and} \qquad T = \begin{pmatrix} 0 & 0 & 0 \\ 1 & 0 & 0 \\ 1 & 0 & 0 \end{pmatrix}. \tag{9}$$

Solutions localized within the unit cell of the termination can generally be written as $|e\rangle = (a, b, c, 0, \cdots)^T$ and must satisfy the matrix equation $H|e\rangle = E_e|e\rangle$, with E_e the energy of the boundary state. This leads to two orthogonal solutions given by

$$|e_1\rangle = \frac{1}{2}\left(\sqrt{2}, 1, -1, 0, \cdots\right)^T \text{ and } |e_2\rangle = \frac{1}{2}\left(\sqrt{2}, -1, 1, 0, \cdots\right)^T, \tag{10}$$

with $E_{e_1} = \sqrt{2}t$ and $E_{e_2} = -\sqrt{2}t$, respectively.

Following the same procedure, we now consider the alternative termination of the chain, shown in Supplementary Figure 2B. The real-space representation of the Hamiltonian is given by

$$H = \begin{pmatrix} D & T^\dagger & 0 & \cdots \\ T & D & T^\dagger & \ddots \\ 0 & T & D & \ddots \\ \vdots & \ddots & \ddots & \ddots \end{pmatrix}. \tag{11}$$

Solving the matrix equation $H|e\rangle = E_e|e\rangle$ for a localized state of the form $|e\rangle = (a, b, c, 0, \cdots)^T$ leads to a single solution

$$|e_0\rangle = \frac{1}{\sqrt{2}}(0, 1, 1, 0, \cdots)^T, \tag{12}$$

with $E_{e_0} = 0$. Importantly, this mode is degenerate with the central bulk band and does not manifest as a topological in-gap state.

Supplementary Note 3

Here we discuss the bulk properties of $H^2(k)$. The spectrum of $H^2(k)$ [cf. Eq. (2) in the main text], has a 2-fold degenerate subspace at energy $2t^2(1 + \cos k + \cos(k - \phi))$ and a single band at zero energy [cf. Fig. 1D in the main text]. In order to properly capture the topological phase of the degenerate bands we use a generalization of Supplementary Eq. (1), called the Wilzcek-Zee phase,

$$\gamma = \int_k^{k+2\pi} \mathrm{Tr}\mathcal{A}(k)\mathrm{d}k, \tag{13}$$

where $\mathcal{A}(k)^{nm} = \langle v_n(k)|\partial_k|v_m(k)\rangle$, and n, m run over the involved states. The bulk solutions of $H^2(k)$ can be written in compact form as

$$|w_1\rangle = \begin{pmatrix} 1 \\ 0 \\ 0 \end{pmatrix}, \quad |w_2\rangle = \frac{1}{\sqrt{2}}\begin{pmatrix} 0 \\ \mathrm{e}^{\mathrm{i}\phi} + \mathrm{e}^{\mathrm{i}k} \\ 1 + \mathrm{e}^{\mathrm{i}k} \end{pmatrix}, \quad |w_3\rangle = \frac{1}{\sqrt{2}}\begin{pmatrix} 0 \\ 1 + \mathrm{e}^{\mathrm{i}k} \\ \mathrm{e}^{\mathrm{i}\phi} + \mathrm{e}^{\mathrm{i}k} \end{pmatrix}, \tag{14}$$

with eigenvalues $2t^2(1 + \cos k + \cos(k - \phi))$, 0 and $2t^2(1 + \cos k + \cos(k - \phi))$, respectively. The squared model, $H^2(k)$, has a band composed of decoupled sites, $|w_1\rangle$, that is degenerate with $|w_3\rangle$. The sub-model, defined by $|w_2\rangle$ and $|w_3\rangle$, is related to an effective Su-Schrieffer-Heeger (SSH) by a rotation $\mathrm{e}^{\mathrm{i}\lambda_3\frac{(\pi-\phi)}{4}}\mathrm{e}^{\mathrm{i}\lambda_2\frac{\pi}{4}}$. At $\phi = \pi$, the resulting effective SSH model is equivalent to a chain with 0 intra-cell coupling, $2t^2$ inter-cell coupling, and a constant $2t^2$ energy shift, see Supplementary Figure 3A, B and C. Using Supplementary Eq. (13) we find

$$\gamma_{1,3} = \pi \bmod 2\pi \quad \text{and} \quad \gamma_2 = \pi \bmod 2\pi. \tag{15}$$

where $\gamma_{1,3}$ denotes the phase of the degenerate subspace spanned by $|w_1\rangle$ and $|w_3\rangle$, and γ_2 is the phase of the single band at zero energy.

The two nonsymmorphic symmetries, Π and χ, obeyed by $H(k)$ are preserved under the squaring operation,

$$\begin{aligned} \Pi H^2(k)\Pi^{-1} &= \left(H^*(k)\right)^2 \,, \\ \chi H^2(k)\chi^{-1} &= \left(H^*(k)\right)^2 \,. \end{aligned} \tag{16}$$

These transformations lead to the following relations between the Wilzcek-Zee phases:

$$\begin{aligned} \Pi: &\quad \gamma_{1,3} \in \{0,\pi\} \quad \text{and} \quad \gamma_2 \in \{0,\pi\}\,, \\ \chi: &\quad \gamma_{1,3} \in \{0,\pi\} \quad \text{and} \quad \gamma_2 \in \{0,\pi\}\,. \end{aligned} \tag{17}$$

Importantly, breaking the χ-symmetry with an onsite potential on the a lattice sites does not make $\gamma_{1,3}$ nor γ_2 lose its quantization. This is because the Π-symmetry protects this quantization.

Supplementary Note 4

In this section we discuss the boundary states of H^2 and their mapping to the boundary states of H. The bulk indices of $H^2(k)$ show that the band polarization of the chain is quantized to nontrivial values. Since the bulk material is insulating, the bulk band polarization manifests as localized states on the boundary [1]. In the following, we give the correspondence between the boundary state found in the spectrum of H (cf. Supplementary Note 2) and the boundary states found in the spectrum of its square, H^2. To this end, we will analyze the two inequivalent lattice termination of H and map those to lattice terminations of H^2.

We start by considering the lattice termination of H given in Supplementary Eq. (8) (cf. Supplementary Figure 2A). Squaring this matrix leads to

$$H^2 = \begin{pmatrix} D^2+TT^\dagger & \{D,T\} & 0 & \cdots \\ \{D,T^\dagger\} & D^2+\{T,T^\dagger\} & \{D,T\} & \ddots \\ 0 & \{D,T^\dagger\} & D^2+\{T,T^\dagger\} & \ddots \\ \vdots & \ddots & \ddots & \ddots \end{pmatrix} , \tag{18}$$

where we have used the fact that $TT = T^\dagger T^\dagger = 0$. The resulting edge termination of H^2 is shown in Supplementary Figure 2A. Importantly, the decoupled state of the boundary unit-cell appears at a lower energy. This is due to the fact that, at the edge, the intra-cell hopping is described by the matrix $D^2 + TT^\dagger$, as opposed to the matrix $D^2 + \{T, T^\dagger\}$ found in the bulk. Localized solutions of the form $|e^2\rangle = (a, b, c, 0, \cdots)^T$ are found by solving the matrix equation $H^2\,|e^2\rangle = E_{e^2}\,|e^2\rangle$. This leads to two orthogonal solutions,

$$|e_1^2\rangle = (1,0,0,0,\cdots)^T \text{ and } |e_2^2\rangle = \frac{1}{\sqrt{2}}\,(0,1,-1,0,\cdots)^T \,, \tag{19}$$

with $E_{e_1^2} = E_{e_2^2} = 2t^2$. We note that $|e_2^2\rangle$ is a topological state related to the standard bulk-edge correspondence of the SSH model, while $|e_1^2\rangle$ appears to be originating from a nontopological inert band. Nevertheless, this distinction is basis-dependent and the appearance of the doubly-degenerate boundary states is a crucial manifestation to our model. Under the square-root operation, the boundary states of H, given in Supplementary Eq. (10), are mapped to linear combinations of $|e_1^2\rangle$ and $|e_2^2\rangle$:

$$|e_1\rangle = \frac{1}{\sqrt{2}}\left(|e_1^2\rangle + |e_2^2\rangle\right) \text{ and } |e_2\rangle = \frac{1}{\sqrt{2}}\left(|e_1^2\rangle - |e_2^2\rangle\right) \,. \tag{20}$$

Following the same procedure as above, we now consider the lattice termination of H given in Supplementary Eq. (11) (cf. Supplementary Figure 2B). This leads to the squared Hamiltonian

$$H^2 = \begin{pmatrix} D^2+T^\dagger T & \{D,T^\dagger\} & 0 & \cdots \\ \{D,T\} & D^2+\{T,T^\dagger\} & \{D,T^\dagger\} & \ddots \\ 0 & \{D,T\} & D^2+\{T,T^\dagger\} & \ddots \\ \vdots & \ddots & \ddots & \ddots \end{pmatrix} . \tag{21}$$

The resulting edge termination of H^2, shown in Supplementary Figure 2B, displays an intra-cell coupling of the form $D^2 + T^\dagger T$. This leads to a localized solution,

$$|e_0^2\rangle = \frac{1}{\sqrt{2}}\left(0, 1, 1, 0, \cdots\right)^T , \tag{22}$$

with energy $E_{e_0^2} = 0$. Comparing with Supplementary Eq. (12), we find that the above state is mapped onto the same state of H:

$$|e_0\rangle = |e_0^2\rangle . \tag{23}$$

In summary, we have provided a relation between the boundary states found in the spectrum of H and H^2. The quantized bulk phases of H^2 lead to a standard topological bulk-edge correspondence, reminiscent of the SSH model, that, under the square root, are mapped to specific boundaries of H. This direct relationship establishes the appearance of protected boundary states in the spectrum of H, despite the fact that the bands below the gap may not have quantized topological indices.

Supplementary Note 5

In this Section, we show a general procedure with which the square root of a standard 2-band 1D TI dressed with an auxiliary state can be taken and discuss extensions to the case where the auxiliary state becomes hybridized. We start by considering non-interacting spinless electrons in a 1-dimensional lattice with three orbital degrees of freedom (d.o.f.) on each lattice site. Assuming that one orbital d.o.f. is completely decoupled from the other two, a general hybridization between the latter two can be written in the basis of 3×3 Hermitian matrices λ_i, with $i = 1, ..., 3$, i.e. the generators of the SU(2) algebra. These are the three Pauli matrices embedded in a 3-dimensional space

$$\lambda_1 = \begin{pmatrix} 0 & 0 \\ 0 & \sigma_x \end{pmatrix} , \quad \lambda_2 = \begin{pmatrix} 0 & 0 \\ 0 & \sigma_y \end{pmatrix} , \quad \lambda_3 = \begin{pmatrix} 0 & 0 \\ 0 & \sigma_z \end{pmatrix} . \tag{24}$$

where together with

$$\lambda_4 = \begin{pmatrix} \sigma_x & 0 \\ 0 & 0 \end{pmatrix} , \quad \lambda_5 = \begin{pmatrix} 0 & 0 & 1 \\ 0 & 0 & 0 \\ 1 & 0 & 0 \end{pmatrix} , \quad \lambda_6 = \begin{pmatrix} \sigma_y & 0 \\ 0 & 0 \end{pmatrix} , \quad \lambda_7 = \begin{pmatrix} 0 & 0 & -\mathrm{i} \\ 0 & 0 & 0 \\ \mathrm{i} & 0 & 0 \end{pmatrix} , \tag{25}$$

and $\lambda_8 = \mathrm{diag}(-2, 1, 1)$ are the eight Gell-Mann matrices. Thus, a general momentum space Hamiltonian is given by

$$H^2(k) = \alpha \begin{pmatrix} 1 & 0 \\ 0 & 0_{2\times 2} \end{pmatrix} + h_0 \begin{pmatrix} 0 & 0 \\ 0 & \mathbb{1}_{2\times 2} \end{pmatrix} + \sum_{i=1}^{3} h_i \lambda_i , \tag{26}$$

where $\mathbb{1}_{2\times 2} = \mathrm{diag}(1, 1)$, $0_{2\times 2} = \mathrm{diag}(0, 0)$, α is the energy at which the decoupled orbital lies, $\mathbf{h} = (h_1, h_2, h_3)$ is a 3-component real-valued vector, and $h_0 = |\mathbf{h}|$ is the energy offset from zero of the two hybridized states. The 1-dimensional topological invariant of the system is encoded in the winding of the Bloch vector $\hat{\mathbf{h}} = \frac{\mathbf{h}}{|\mathbf{h}|}$, defined as

$$w = \frac{1}{2} \int\limits_{\mathrm{BZ}} \mathrm{d}k \hat{\mathbf{h}} \times \partial_k \hat{\mathbf{h}} . \tag{27}$$

A nontrivial TI has a Bloch vector constrained to move in a specific plane in the SU(2) subspace and its trajectory, as one moves through the 1-dimensional BZ, traces a circle. This leads to a nontrivial π winding in the bulk and mid-gap states localized on the boundary [1]. Such constraint implies that one of the components of $\hat{\mathbf{h}}$ is zero and that $H^2(k)$ has a chiral-like symmetry, Θ, that anticommutes with the 2×2 subblock, $\sum_{i=1}^{3} h_i \lambda_i$. As long as the 2×2 subblock of hybridized states remains decoupled from the remaining itinerary state and Θ is not broken by any perturbation or disorder, the boundary states remain topologically protected, in similitude to standard chiral 1D TIs.

In order to define the square-root operation, we need to find a self adjoint Hamiltonian $\sqrt{H^2}(k)$ that squares to Supplementary Eq. (26). Using the properties of the $SU(3)$ algebra, the most general self adjoint Hamiltonian that squares to Supplementary Eq. (26) is given by

$$\sqrt{H^2}(k) = \sum_{i=1}^{4} d_i \lambda_{i+3} , \tag{28}$$

where $\mathbf{d} = (d_1, d_2, d_3, d_4)$ is a 4-component real-valued vector. The square of $\sqrt{H^2}(k)$, given by

$$\left(\sqrt{H^2}(k)\right)^2 = \sum_{i=1}^{4} d_i^2 \lambda_{i+3}^2 + \sum_{i<j}^{4} d_i d_j \{\lambda_{i+3}, \lambda_{j+3}\} \equiv H^2(k)\,, \tag{29}$$

defines a relation between the components of $\mathbf{d}$ and $\mathbf{h}$, namely

$$\begin{aligned} 2h_0 &= \mathbf{d}\cdot\mathbf{d} = \alpha\,, \\ h_1 &= d_1 d_3 + d_2 d_4\,, \\ h_2 &= d_1 d_4 - d_3 d_2\,, \\ h_3 &= \frac{(d_1^2 + d_2^2 - d_3^2 - d_4^2)}{2}\,, \end{aligned} \tag{30}$$

We note that the above equations do not have a unique solution. Therefore, a given $\mathbf{h}$ can have different square root realizations. In fact, any unitary transformation of $\sqrt{H^2}(k)$ has no effect on the resulting $H^2(k)$.

If additional terms proportional to the remaining Gell-Mann matrices are included in Supplementary Eq. (28) the squared model will have all three orbits hybridized. Nonetheless, if there exists a symmetry that quantizes $\gamma_{1,3}$ and γ_2 in the same way as in Supplementary Eq. (17), the resulting squared model will be a topological insulator with (in the nontrivial case) protected in-gap boundary states. For example, an onsite potential d on the a site of the AB cages introduces terms proportional to λ_i, where $i = \{4, 5, 6, 7\}$, to the squared Hamiltonian $H^2(k)$. However, such on site potential term preserves the Π-symmetry and breaks the χ-symmetry. As a result, the squared Hamiltonian at 1/3-filling has quantized winding numbers and topologically protected in-gap boundary states. In the square root model, these states are found above bands with nonquantized phases [cf. Supplementary Eq. (7)], yet remain robust against additional disorder that does not break the quantization in the squared model $H^2(k)$ (cf. Supplementary Note 6 and Supplementary Note 7).

Going back to our original model, the AB-cages at $\phi = \pi$, we find that $H^2(k)$ has $h_1 = 0$ and, therefore, the subblock $\sum_{i=1}^{3} h_i \lambda_i$ has a chiral symmetry given by $\Theta = \mathrm{e}^{\mathrm{i}\frac{\pi}{2}\lambda_1}$, that quantizes the topological phases to

$$\Theta: \qquad \gamma_{1,3} \in \{0, \pi\} \qquad \text{and} \qquad \gamma_2 \in \{0, \pi\}\,, \tag{31}$$

where $\gamma_{1,3}$ denotes the phase of the degenerate subspace, and γ_2 is the phase of the single band at zero energy. Such symmetry is preserved when the AB-cages model is of the form of Supplementary Eq. (28), i.e., there are no additional terms proportional to the remaining Gell-Mann matrices, and the hopping strength between sites is equal. In this case, the topological boundary states appear in the middle of the gap and are pinned to their energy due to the chiral symmetry Θ.

For completeness, we note that different gauge choices of the AB-cages lead to an equivalent squared model. This has to do with the fact that transformations which preserve the boundary conditions have vanishing contribution to the topological invariant. Therefore, the Bloch vector, $\mathbf{h}$, will still correspond to a nontrivial TI that winds around a circle as we go around the 1-dimensional BZ. Conversely, the two alternative unit-cell choices of the AB-cages that we did not consider here lead to a Bloch vector $\mathbf{h}$ that does not correspond to a standard TI.

Supplementary Note 6

In this section we discuss the interplay of an onsite potential with the Π-preserving term. Adding an onsite potential of the form

$$H(k) = \begin{pmatrix} d & t + t\mathrm{e}^{-\mathrm{i}k} & t\mathrm{e}^{-\mathrm{i}\phi} + t\mathrm{e}^{-\mathrm{i}k} \\ t + t\mathrm{e}^{\mathrm{i}k} & 0 & 0 \\ t\mathrm{e}^{\mathrm{i}\phi} + t\mathrm{e}^{\mathrm{i}k} & 0 & 0 \end{pmatrix}, \tag{32}$$

leads to scrambling of winding phases γ_1 and γ_3, while γ_2 remains quantized, see Supplementary Figure 4 [cf. Supplementary Eq. (7)]. Importantly, the boundary states given in Supplementary Eq. (10) remain solutions of the Hamiltonian but with modified energies

$$E_{e_1} = \frac{1}{2}\left(d + \sqrt{d^2 + 8}\right) \text{ and } E_{e_2} = \frac{1}{2}\left(d - \sqrt{d^2 + 8}\right)\,, \tag{33}$$

while the boundary state given in Supplementary Eq. (12) remains pinned to zero energy, i.e., it does not manifest as a topological in-gap state.

The robustness of the boundary states against additional disorder (cf. Supplementary Figs. 5 and 6) stems from the fact that the Π-transformation [cf. Supplementary Eq. (6)] remains a symmetry of both $H(k)$ and $H^2(k)$, while the χ-symmetry is broken. Since the quantization of the Wilzcek-Zee phases persists under the addition of this term [cf. Supplementary Eq. (17)], the localization of the boundary states is unaffected (cf. Supplementary Note 7 and Supplementary Figs. 5 and 6).

Supplementary Note 7

One of the fundamental aspects of topological insulators is the robustness of their boundary states against disorder that does not break the symmetry that quantizes the topological invariant. Such a property is paramount towards realizing fault-tolerant and impurity-insensitive devices. In this Section, we investigate the robustness of the boundary states found in the AB-cages against different kinds of disorder.

The topological robustness of boundary states is commonly quantified by calculating the energy offset of the topological state as well as its localization length over many disorder realizations. For a comprehensive analysis, we study, on one hand, (a) disorder that on average preserves both symmetries, Π and χ, and on the other hand (b) terms that on average break each of these symmetries individually and jointly and analyze the impact of additional disorder on top of the symmetry-broken model. The analyzed quantities are the averaged mean squared difference of the boundary state energy in the presence of disorder, β_{edge}, from the energy of the undisturbed system, β_0 (i.e. when $\sigma = 0$) and the second moment of the corresponding eigenstates. The former shows the robustness of the energy while the latter quantifies the localization of the boundary state and is related to the localization length. The disorder is assumed to be of Gaussian shape with standard deviation σ and a vanishing mean for case (a) or a mean equal to the coupling strength t for the case (b). Each studied disorder strength was average over 10000 realizations on a lattice with 99 sites.

In order to analyze the ensuing impact of symmetry-breaking terms and disorder, we consider three different types, namely, potential terms d on sites a, and complex coupling terms α on the hopping amplitudes between sites b and c

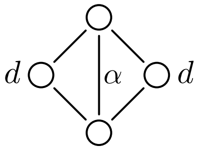

(34)

The corresponding symmetries that survive under the addition of such terms are

$$\begin{array}{ccc} \text{Term} & \text{Preserved} & \text{Broken} \\ d & \Pi & \chi\,, \\ \text{Re}(\alpha) & \chi & \Pi\,. \end{array} \tag{35}$$

The results are displayed in Supplementary Figs. 5 and 6 for vanishing mean and non-vanishing mean disorder realizations, respectively. A Π-preserving, but χ-breaking, disorder is implemented by adding a real detuning term d that on average is either $\langle d \rangle = 0$ or t (see black curve in Supplementary Figs. 5 and 6). A χ-preserving, but Π-breaking, disorder is implemented by adding a real coupling term α that on average is either $\langle \alpha \rangle = 0$ or t (see red curve in Supplementary Figs. 5 and 6). A general disorder that breaks all symmetries is implemented by combining a complex coupling α and a detuning d (see blue curve in Supplementary Figs. 5 and 6 for $\langle \alpha \rangle = \langle d \rangle = 0$ and $\text{Re}(\langle \alpha \rangle) = \text{Im}(\langle \alpha \rangle) = \langle d \rangle = t$, respectively). We illustrate the robustness of the edge state energies more clearly, by splitting the plot into two regions with an energy offset that is bigger (red) or smaller (green) than $(\sigma/t)^2$, since $(\sigma/t)^2$ would correspond to a an energy offset proportional to the disorder strength.

We find that, indeed, in case of zero average disorder, the boundary modes remain robust in both energy and localization length against a symmetry-preserving disorder and as long as the disorder strength is not large enough to mix the boundary states with the nearest bulk band. In the case of either Π- or χ-preserving disorder we find that the energy offset is always in the green region (see Supplementary Figure 5a), while the wavefunctions remain highly localized within a unit cell (see Supplementary Figure 5b). In contrast, the boundary states do not show any

protection against a general disorder, neither in energy, since we find that the energy offset is mostly in the red region (see Supplementary Figure 5a), nor localization, even though the symmetries are preserved on average. For nonzero average disorder (i.e. adding a nonzero symmetry-breaking term to the Hamiltonian and applying an additional disorder), the boundary states still appear, but are now pinned to a different energy, other than $\pm\sqrt{2}t$. This is due to the fact that the square-root map between the effective-SSH and the AB-cages model no longer holds. Nevertheless, the remaining unbroken symmetries in the modified AB-cages model still provide protected boundary states since the corresponding squared model is again a nontrivial TI (see Supplementary Figure 6).

Supplementary Note 8

In this Section, we provide additional information on the effective negative hopping between two main waveguides when an auxiliary waveguide is introduced between them. The auxiliary waveguide is strongly detuned from the main waveguides and, hence, inhibits direct coupling between them. Moreover, it is generating an effective negative hopping between the main waveguides through a carefully-tuned second-order tunneling process, i.e., this approach allows us to effectively flip the sign of the coupling amplitude between them. Embedding such a negative hopping amplitude inside the square structure of our system realizes an artificial gauge field of a π flux for the photons, i.e., when a particle goes around the square it collects the negative sign which is equivalent to a $\mathrm{e}^{\mathrm{i}\pi}$ phase.

For completeness, we provide here a more detailed description of such negative hopping generation (cf. also Ref. [2]). We start by considering two main waveguides with a small detuning δ, coupled to each other via the overlap of evanescence modes with a neighbouring auxiliary waveguide with detuning Δ. Using the tight-binding approximation, the equations of motions for the electric-field amplitude in the three waveguides, a, b and c, are given by

$$\mathrm{i}\partial_z \begin{pmatrix} a \\ b \\ c \end{pmatrix} = \begin{pmatrix} \delta & \kappa & 0 \\ \kappa & \Delta & \kappa \\ 0 & \kappa & \delta \end{pmatrix} \begin{pmatrix} a \\ b \\ c \end{pmatrix},$$

where κ is the coupling strength. In order to engineer the negative coupling between the two main waveguides, a and c, we choose

$$\Delta = \frac{\kappa^2 - \delta^2}{\delta},$$

and find the eigenvectors and eigenvalues

$$\mathbf{u}_1 = \begin{pmatrix} 1 \\ 0 \\ -1 \end{pmatrix} \qquad \beta_1 = \delta$$

$$\mathbf{u}_2 = \begin{pmatrix} 1 \\ -2\dfrac{\delta}{\kappa} \\ 1 \end{pmatrix} \qquad \beta_2 = -\delta$$

$$\mathbf{u}_3 = \begin{pmatrix} 1 \\ \kappa/\delta \\ 1 \end{pmatrix} \qquad \beta_3 = \delta + \frac{\kappa^2}{\delta}\,.$$

In the limit where $\delta \ll \kappa$ the eigenvectors can be approximated by

$$\tilde{\mathbf{u}}_1 = \begin{pmatrix} 1 \\ 0 \\ -1 \end{pmatrix} \qquad \tilde{\mathbf{u}}_2 \approx \begin{pmatrix} 1 \\ 0 \\ 1 \end{pmatrix} \qquad \tilde{\mathbf{u}}_3 \approx \begin{pmatrix} 0 \\ \kappa/\delta \\ 0 \end{pmatrix},$$

and the eigenvalues by

$$\beta_1 = \delta\,, \quad \beta_2 = -\delta\,, \quad \beta_3 \approx \frac{\kappa^2}{\delta}\,,$$

respectively. Since $\tilde{\mathbf{u}}_3$ is decoupled from the remaining two states and its eigenvalue is much larger, its dynamics will not contribute much to the evolution of amplitudes a and c, hence it can be traced out. The effective model obtained

after tracing out the auxiliary waveguide b is equivalent to having two waveguides that are coupled with a negative amplitude, i.e.,

$$\mathrm{i}\partial_z \begin{pmatrix} a \\ c \end{pmatrix} = \begin{pmatrix} 0 & -\delta \\ -\delta & 0 \end{pmatrix} \begin{pmatrix} a \\ c \end{pmatrix} .$$

One can see by direct calculation that the eigenvalues and eigenvectors of the above Hamiltonian are equivalent to $\tilde{\mathbf{u}}_1$ and $\tilde{\mathbf{u}}_3$ with eigenvalues δ and $-\delta$ respectively. Embedding such an effective model inside the square structure of our AB-cages system and carefully choosing the detuning δ such that it matches the coupling strength between waveguides on the other edges of the square, realizes an artificial gauge field with π flux threading each square.

Supplementary Note 9

In this section we discuss the propagation without flux. In order to demonstrate that the edge states, as well as the complete localisation of the bulk states originate from the flux of $\phi = \pi$ we also fabricated a lattice without flux and measured the resulting intensities (see Supplementary Figure 7). It can clearly be seen that for the case of vanishing flux no localisation occurs when exciting the bulk (see Supplementary Figure 7a-b). When exciting the edge of the lattice there is basically no light residing, which confirms that there is no edge state present (see Supplementary Figure 7c).

SUPPLEMENTARY REFERENCES

[1] Resta, R. & Vanderbilt, D. *Physics of Ferroelectrics.* (Springer, Berlin, 2007).
[2] Keil, R. et al. Universal sign control of coupling in tight-binding lattices. *Physical Review Letters* **116**, 213901-1–213901-5 (2016).
